# Competition-Based Success Factors During the Talent Pathway of Elite Male Swimmers

**DOI:** 10.3389/fspor.2020.589938

**Published:** 2020-11-20

**Authors:** Dennis-Peter Born, Ishbel Lomax, Stephan Horvath, Elena Meisser, Philipp Seidenschwarz, David Burkhardt, Michael Romann

**Affiliations:** ^1^Department for Elite Sport, Swiss Federal Institute of Sport Magglingen, Magglingen, Switzerland; ^2^Swiss Swimming Federation, Bern, Switzerland; ^3^College of Life and Environmental Sciences, University of Exeter, Exeter, United Kingdom; ^4^Centre of Technologies in Sports and Medicine, Bern University of Applied Sciences, Nidau-Biel, Switzerland; ^5^Department of Mathematics and Computer Science, University of Basel, Basel, Switzerland

**Keywords:** age, key performance indicators, long-term athlete development, number of races, performance variation

## Abstract

Marginal differences in race results between top swimmers have evoked the interest in competition-based success factors of long-term athlete development. To identify novel factors for the multi-dimensional model of talent development, the aim of the study was to investigate annual variation in competition performance (ACV), number of races per year, and age. Therefore, 45,398 race results of all male participants (*n* = 353) competing in individual events, i.e., butterfly, backstroke, breaststroke, freestyle, and individual medley, at the 2018 European Long-Course Swimming Championships (2018EC) were analyzed retrospectively for all 10 years prior to the championships with Pearson's correlation coefficient and multiple linear regression analysis. Higher ranked swimmers at the 2018EC showed significant medium correlations with a greater number of races per year and small but significant correlations with higher ACV in 10 and nine consecutive years, respectively, prior to the championships. Additionally, better swimmers were older than their lower ranked peers (*r* = −0.21, *p* < 0.001). Regression model explained a significant proportion of 2018EC ranking for 50 m (47%), 100 m (45%), 200 m (31%), and 400 m races (29%) but not for 800 and 1,500 m races with number of races having the largest effect followed by age and ACV. In conclusion, higher performance variation with results off the personal best in some races did not impair success at the season's main event and young competitors at international championships may benefit from success chances that increase with age. The higher number of races swum per year throughout the career of higher ranked swimmers may have provided learning opportunities and specific adaptations. Future studies should quantify these success factors in a multi-dimensional talent development model.

## Introduction

The men's recent Olympic 50 m freestyle final was won by only a 100th of a second (IOC, 2016a). Such marginal differences in race results of top swimmers have evoked interest in success factors at all competition levels, from elite to junior swimmers (Stewart and Hopkins, 2000; Pyne et al., 2004; Post et al., 2020). For scientific and longitudinal race analyses, Olympic pool swimming provides highly standardized conditions, due to FINA (Fédération International de Natation) rules (FINA, 2020b). These only allow for a tolerance of +0.01 m for pool length and specify that the current must be below 1.25 m per 60 s (FINA, 2020b). Furthermore, electronical time keeping is compulsory (FINA, 2020b). Therefore, comparison of results across various venues and championships over several years is feasible and variations in performance can be determined.

At a junior level for instance, faster swimmers showed a lower performance variation (1.1%) between two important competitions than their slower counterparts (1.5%) (Stewart and Hopkins, 2000). At elite-level, variation in competition performance decreased in the 5 years leading up to the Olympics (Costa et al., 2010; Clephas and Wilhelm, 2019). Furthermore, performance variation increased from short-distance (0.5%) to long-distance (1.0%) events (Pyne et al., 2004), while differences between swimming strokes remained unclear (Pyne et al., 2004; Trewin et al., 2004). However, at major competitions, i.e., World Championships and Olympic games, higher performance variation that improve current seasonal best times was an important contributor to success of finalists (Mujika et al., 2019). With these conflicting data, practical application of performance variation in competitive swimming is limited. Lack of significant findings or presentation of trend-like effects warrant further investigations, in particular on performance variation during the development process of young talented swimmers.

Top-level Olympic swimmers competing in long-distance freestyle events reached peak performance at a younger age (22.9 ± 2.2 yrs) than the competitors in freestyle sprint events (25.9 ± 1.9 yrs). However, there was no difference in the window of peak performance, i.e., 2.9 ± 1.5 and 3.1 ± 1.8 yrs, respectively, between freestyle long-distance and sprint swimmers (Allen et al., 2014). While achievement of top performances might result from multiple factors, i.e., genetics, economies, and available support (Tucker and Collins, 2012; Breitbach et al., 2014), there is a heated debate about the effect of age and the associated accumulation of practice (Ericsson et al., 1993; Tucker and Collins, 2012; Ericsson and Harwell, 2019). Therefore, the effect of age on final ranking at international competitions is of further interest. In addition, a recent study examining swimmers showed that the number of years competing at a high international level is an important success factor for upcoming competitions (Yustres et al., 2017). From a physiological and psychological perspective, multiple competitions might help develop race routines and resilience (Carrasco et al., 2007; Meggs et al., 2016; Burns et al., 2019). However, little is known about the optimal number of races over the course of the season, or how it affects performance variation and long-term athlete development.

Therefore, the aim of this study was to identify novel competition-based success factors and investigate annual variation in competition performance (ACV), number of races per year, and age for all 10 years prior to the 2018EC. It was hypothesized that better swimmers at the 2018EC would (1) show more consistent performance and lower ACV, (2) participate in more races per year, and (3) be older than athletes at the bottom of the 2018EC ranking.

## Materials and Methods

### Participants

Race results of all male participants (*n* = 353, age 22.5 ± 3.2 yrs) competing in individual events at the 2018EC in Glasgow were analyzed retrospectively for all 10 years preceding the championships. In total, 45,398 races were analyzed. Pearson's correlation coefficient and multiple regression analysis were used to analyze the relationship between ranking achieved at the 2018EC as the dependent variable and potential success factors i.e., ACV, number of races per year, and age. The study was approved by the leading institution's review board (Reg.-Nr. 088LSP250919) and is in the spirit of the Code of Ethics of the World Medical Association (Helsinki Declaration).

### Data Collection

For the present study, data were provided by the publicly accessible domain “swimrankings.net,” which displays race results from the official database of LEN (Ligue Européenne de Natation), the governing European swimming federation. The database lists results from registered races which are in accordance with official FINA (Fédération Internationale de Natation) rules (FINA, 2020b), including electronical time keeping and limits to in-pool current. As the European championships are the highest international competition of the LEN, the 2018EC ranking was chosen as the dependent variable. Written informed consent was provided by the domain's owner for anonymized usage and publication of the data.

### Data Analysis

Competition results of long-course competitions were analyzed for ACV and number of races per year. Swimmers' annual ACVs were determined as the coefficient of variance (standard deviation divided by the mean) for each swimming stroke and race distance they performed in every year back to 2009. A minimum of two races per event per year were required to calculate the coefficient of variance and to include data in the regression model. Number of races were determined for each year, stroke, and distance. Age in the year of the 2018EC was added as third independent variable to the model.

Previous studies showed an improvement of 3–4% in performance in the five seasons leading up to the Olympics (Costa et al., 2010). For the present study, data were analyzed with no information on tapering, pacing strategies, injuries, or illnesses. No information was available on importance of the races within the competition schedule. In less important races, athletes lying far behind might have given up the race and finished beyond a functional variation in competition performance. Therefore, outliers were defined as coefficients of variance showing an ACV >4% within 1 year. This led to the exclusion of 113 out of 5'555 calculated coefficients.

### Statistical Analysis

Athletes' ACV and number of races per year (2018–2009) were correlated with their 2018EC ranking using Pearson's correlation coefficient for an initial descriptive analysis. Correlation coefficients of 0.1–0.3, 0.3–0.5, and 0.5–0.7 were classified as small, medium, and large (Hopkins, 2002). Since butterfly, backstroke, and breaststroke events involve race distances up to 200 m only, swimming strokes were compared using pooled data of the 50, 100, and 200 m events. Freestyle events were used for the comparison between race distances (50, 100, 200, 400, 800, and 1,500 m). Using multiple linear regression analysis, predictive values were determined with ACV, number of races per year, and age for the final 2018EC ranking. Mean values of the last 2 years before the 2018EC were applied in the regression model. An alpha level of <0.05 confirmed a statistically significant effect. Based on standard procedure for multiple regression analysis on large sample sizes, normality was investigated on the standardized residuals and predicted values (Field, 2013). In scatterplots, data were evenly distributed in a random pattern around zero. Histograms showed a Gaussian distribution of the regression standardized residuals and normal probability plots showed a diagonal straight line confirming normally distributed data (Field, 2013). Data were collected and prepared with Microsoft Excel 2016 (Microsoft Corporation, Redmond, WA, USA). Analysis were carried out using SPSS statistical software package for Windows Version 25.0 (IBM Corporation, Armonk, NY, USA).

## Results

### Descriptive Statistics

**Figure 1:**
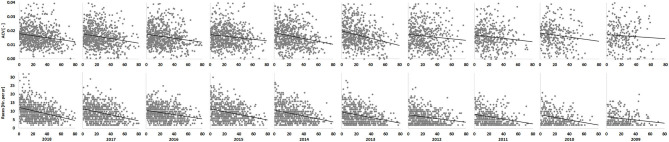
Correlation matrixes of all swimming strokes and race distances for annual variation in competition performance (ACV) and number of races per year (Races). Data points represent individual values for each participant and year in regard to ranking at the 2018 European Swimming Championships.

**Figure 2:**
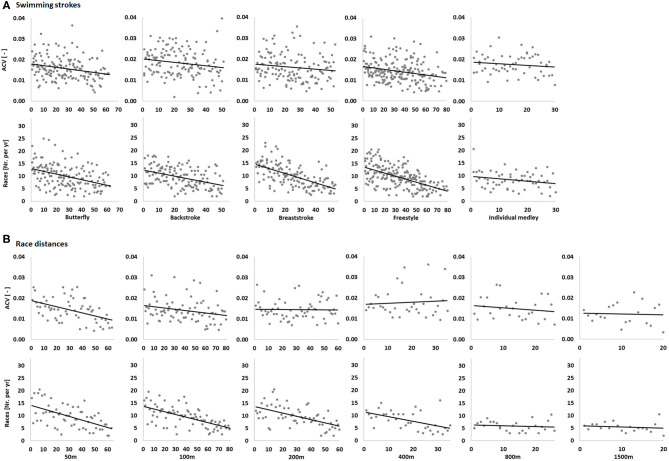
Correlation matrixes of each swimming stroke **(A)** and race distance **(B)** for annual variation in competition performance (ACV) and number of races per year (Races). Data points represent individual values for each participant in regard to ranking at the 2018 European Swimming Championships.

Correlations between ranking at the 2018EC and ACV and number of races per year are presented in Tables 1, 2 as well as Figures 1, 2. Pooled data of all swimming strokes and race distances showed that higher ranked swimmers at the 2018EC had a larger ACV for nine consecutive years prior to the championships, compared to their lower ranked counterparts. More variation was also evident for the years that immediately preceded the championships for butterfly and freestyle as well as the 50 m and 100 m events.

**Table 1:** Annual variation in competition performance (mean ± standard deviation) correlated with ranking at the 2018 European Swimming Championships.

	**2018**	**2017**	**2016**	**2015**	**2014**	**2013**	**2012**	**2011**	**2010**	**2009**
All strokes and distances	1.6 ± 0.7%	1.6 ± 0.7%	1.6 ± 0.7%	1.6 ± 0.7%	1.6 ± 0.8%	1.7 ± 0.8%	1.6 ± 0.8%	1.6 ± 0.8%	1.7 ± 0.8%	1.7 ± 0.9%
	−0.18***	−0.19***	−0.17***	−0.13***	−0.22***	−0.26***	−0.11*	−0.13*	−0.13*	*−0.06*
Butterfly	1.6 ± 0.6%	1.6 ± 0.7%	1.6 ± 0.7%	1.6 ± 0.7%	1.6 ± 0.8%	1.6 ± 0.8%	1.7 ± 1.0%	1.8 ± 0.7%	1.7 ± 0.8%	2.0 ± 1.0%
	−0.22**	−0.17*	−0.25**	*−0.13*	*−0.12*	−0.37***	*−0.18*	*−0.18*	*−0.21*	*0.06*
Backstroke	1.8 ± 0.7%	1.8 ± 0.7%	1.8 ± 0.7%	1.7 ± 0.8%	1.8 ± 0.9%	1.9 ± 0.8%	1.7 ± 0.9%	1.5 ± 0.7%	1.6 ± 0.9%	1.7 ± 0.9%
	*−0.12*	−0.27**	−0.26**	*−0.12*	−0.28**	−0.21*	−0.24*	*−0.23*	*−0.17*	*−0.26*
Breaststroke	1.6 ± 0.7%	1.6 ± 0.8%	1.6 ± 0.7%	1.6 ± 0.7%	1.5 ± 0.8%	1.6 ± 0.8%	1.6 ± 0.9%	1.6 ± 0.9%	1.9 ± 1.0%	1.6 ± 1.0%
	*−0.04*	−0.22*	*0.01*	*−0.13*	*−0.15*	−0.24*	*−0.19*	*−0.04*	*−0.14*	*0.02*
Freestyle	1.4 ± 0.6%	1.4 ± 0.7%	1.4 ± 0.7%	1.5 ± 0.6%	1.4 ± 0.6%	1.4 ± 0.7%	1.5 ± 0.8%	1.6 ± 0.7%	1.6 ± 0.7%	1.4 ± 0.8%
	−0.25***	−0.17*	*−0.10*	*−0.11*	*−0.13*	*−0.10*	*0.05*	−0.33**	*−0.10*	*−0.11*
Individual medley	1.8 ± 0.5%	2.1 ± 0.7%	2.0 ± 0.8%	2.0 ± 0.5%	2.0 ± 0.8%	2.3 ± 1.0%	2.1 ± 0.8%	1.5 ± 0.9%	1.8 ± 0.6%	1.7 ± 0.7%
	*−0.05*	*0.02*	*0.27*	*0.30*	*0.04*	*−0.39*	*−0.17*	*−0.34*	*−0.14*	*0.05*
50 m	1.5 ± 0.6%	1.4 ± 0.7%	1.6 ± 0.8%	1.6 ± 0.7%	1.3 ± 0.6%	1.2 ± 0.6%	1.4 ± 0.8%	1.5 ± 0.8%	1.5 ± 0.8%	1.1 ± 0.8%
	−0.43***	−0.35*	*−0.03*	*−0.12*	*−0.23*	*−0.05*	*0.17*	*−0.32*	*−0.28*	*−0.44*
100 m	1.4 ± 0.6%	1.4 ± 0.7%	1.2 ± 0.5%	1.4 ± 0.6%	1.4 ± 0.7%	1.5 ± 0.6%	1.4 ± 0.7%	1.5 ± 0.7%	1.5 ± 0.7%	1.5 ± 0.7%
	−0.33*	*−0.09*	−0.21*	−0.16**	−0.06*	−0.19**	*0.08*	*−0.25*	*−0.01*	*−0.08*
200 m	1.5 ± 0.5%	1.5 ± 0.7%	1.4 ± 0.6%	1.5 ± 0.5%	1.5 ± 0.5%	1.6 ± 0.9%	1.7 ± 0.9%	1.7 ± 0.6%	1.9 ± 0.8%	1.7 ± 1.0%
	*0.16*	*−0.16*	*0.12*	*0.08*	*−0.11*	*−0.12*	*−0.04*	−0.58**	*−0.05*	*−0.22*
400 m	1.7 ± 0.8%	1.7 ± 0.6%	1.7 ± 0.6%	1.6 ± 0.6%	2.2 ± 0.7%	2.4 ± 0.8%	1.9 ± 0.7%	1.9 ± 0.8%	1.8 ± 0.7%	1.6 ± 0.9%
	*0.00*	*−0.01*	*−0.22*	*−0.33*	*−0.23*	−0.41*	*0.01*	*0.46*	*0.23*	*0.12*
800 m	1.5 ± 0.6%	1.5 ± 0.7%	1.5 ± 0.6%	1.7 ± 0.7%	1.9 ± 0.7%	1.8 ± 1.0%	1.5 ± 0.6%	1.2 ± 0.7%	1.9 ± 1.0%	1.3 ± 0.9%
	*−0.19*	*−0.01*	*0.19*	*0.28*	*−0.37*	*−0.05*	*0.40*	*0.50*	*−0.31*	*−0.17*
1,500 m	1.4 ± 0.8%	1.1 ± 0.6%	1.3 ± 0.4%	1.4 ± 0.8%	1.7 ± 0.8%	1.4 ± 0.7%	1.2 ± 0.4%	1.3 ± 0.7%	1.8 ± 0.8%	1.5 ± 0.7%
	*−0.06*	*0.10*	*0.08*	*0.22*	*−0.06*	*−0.21*	*−0.02*	*−0.42*	*−0.10*	*−0.51*

*p < 0.05.

**p < 0.01.

***p < 0.001.

*Pearson's correlation coefficient (r) with corresponding statistical significance is indicated in italic letters below the descriptive data for all 10 years prior to the championships*.

**Table 2:** Number of races per year (mean ± standard deviation) correlated with ranking at the 2018 European Swimming Championships.

	**2018**	**2017**	**2016**	**2015**	**2014**	**2013**	**2012**	**2011**	**2010**	**2009**
All strokes and distances	9.8 ± 4.9	9.3 ± 4.7	9.0 ± 4.3	9.4 ± 4.9	8.6 ± 4.9	7.7 ± 4.6	6.7 ± 4.1	6.6 ± 4.3	6.4 ± 4.2	5.8 ± 3.8
	−0.33***	−0.31***	−0.25***	−0.28***	−0.29***	−0.29***	−0.23***	−0.26***	−0.27***	−0.19**
Butterfly	10.6 ± 5.3	10.0 ± 5.1	8.4 ± 4.2	9.0 ± 5.3	7.7 ± 4.7	7.2 ± 4.3	5.9 ± 3.4	6.9 ± 4.6	6.1 ± 4.0	6.2 ± 3.8
	−0.35***	−0.33***	−0.24**	−0.33***	−0.41***	−0.24*	−0.30**	−0.45***	−0.32*	*−0.02*
Backstroke	9.6 ± 4.4	10.0 ± 4.3	9.9 ± 4.8	9.9 ± 5.2	10.1 ± 5.2	8.3 ± 4.8	6.7 ± 3.8	6.6 ± 4.8	7.2 ± 5.2	6.3 ± 4.2
	−0.48***	−0.25**	−0.28**	−0.33***	−0.39***	−0.32***	−0.27*	−0.36**	−0.43**	−0.37*
Breaststroke	10.7 ± 5.3	9.7 ± 5.1	9.8 ± 4.2	9.9 ± 5.0	8.5 ± 4.9	7.9 ± 4.3	6.8 ± 3.9	6.2 ± 3.8	6.6 ± 3.9	6.8 ± 4.6
	−0.60***	−0.46***	−0.34***	−0.31***	−0.25**	−0.37***	−0.28**	−0.22*	−0.32**	−0.30*
Freestyle	10.3 ± 5.0	9.1 ± 4.8	9.2 ± 4.2	9.7 ± 4.8	8.8 ± 5.0	7.2 ± 4.3	6.6 ± 3.9	6.9 ± 4.2	6.2 ± 4.2	4.9 ± 2.9
	−0.49***	−0.48***	−0.37***	−0.42***	−0.27***	−0.26**	*−0.14*	−0.27*	−0.28*	*−0.25*
Individual medley	9.6 ± 4.2	9.7 ± 4.4	10.5 ± 4.0	10.5 ± 4.9	9.2 ± 4.4	8.1 ± 4.0	7.1 ± 4.3	7.3 ± 4.4	7.6 ± 4.4	5.4 ± 3.5
	*−0.25*	*−0.33*	*−0.38*	−0.42*	−0.70***	−0.58**	−0.52*	−0.73***	*−0.41*	*−0.24*
50 m	10.2 ± 5.6	9.5 ± 5.1	9.1 ± 4.4	9.6 ± 4.7	8.6 ± 5.3	6.8 ± 4.5	7.1 ± 3.9	7.4 ± 5.0	5.8 ± 4.9	5.2 ± 3.8
	−0.49***	−0.39**	−0.37**	−0.40**	−0.42**	−0.43**	−0.42**	−0.57**	−0.47*	*−0.28*
100 m	10.0 ± 4.7	9.1 ± 4.7	9.2 ± 4.5	9.8 ± 5.2	9.6 ± 5.2	7.6 ± 4.2	7.0 ± 3.9	7.0 ± 4.3	6.9 ± 4.3	5.2 ± 2.7
	−0.55***	−0.50***	−0.46***	−0.48***	−0.29*	−0.26*	*−0.04*	*−0.23*	*−0.28*	*−0.25*
200 m	10.8 ± 4.7	8.7 ± 4.5	9.4 ± 3.6	9.9 ± 4.3	8.0 ± 4.4	7.0 ± 4.3	5.4 ± 3.5	6.3 ± 3.2	5.3 ± 2.9	3.6 ± 1.6
	−0.43**	−0.60***	*−0.22*	−0.39**	*−0.17*	*−0.14*	*−0.09*	*−0.08*	*−0.00*	*−0.34*
400 m	8.1 ± 4.0	8.5 ± 4.1	8.3 ± 3.5	8.8 ± 3.7	10.7 ± 5.7	12.1 ± 7.7	10.8 ± 7.6	9.4 ± 5.5	7.9 ± 4.8	5.8 ± 4.4
	−0.41*	−0.48**	−0.59**	−0.47**	−0.73***	−0.65***	*−0.42*	*0.44*	*−0.05*	*−0.08*
800 m	6.0 ± 2.1	5.9 ± 2.6	5.8 ± 2.2	7.1 ± 3.4	6.6 ± 1.8	6.4 ± 3.9	6.7 ± 3.1	5.4 ± 3.2	5.0 ± 3.0	4.0 ± 2.4
	*−0.03*	*−0.14*	*−0.24*	*−0.04*	*−0.23*	−0.50 *	*−0.18*	*−0.15*	*0.21*	*−0.64*
1,500 m	5.4 ± 2.1	5.6 ± 2.3	6.1 ± 1.8	7.0 ± 3.2	5.1 ± 1.7	5.8 ± 2.3	5.7 ± 2.6	4.4 ± 2.2	4.5 ± 1.8	4.3 ± 1.5
	*−0.06*	*−0.06*	*−0.25*	*0.19*	*−0.09*	−0.67**	*−0.30*	*−0.11*	*−0.39*	*−0.28*

*p < 0.05.

**p < 0.01.

***p < 0.001.

*Pearson's correlation coefficient (r) with corresponding statistical significance are indicated in italic letters below the descriptive data for all 10 years prior to the championships*.

The pooled data of all swimming strokes and race distances showed that higher 2018EC ranking was related to more races per year during 10 consecutive years prior to the championships (refer to Table 2). The more detailed analyses showed that this was the case for all swimming strokes, but less clear for individual medley. Furthermore, a higher 2018EC ranking was associated with more races per year in the 50, 100, 200, and 400 m events. Finally, age was related to success, with older swimmers ranking higher at the 2018EC (*r* = −0.21, *p* < 0.001). Regarding swimming strokes, age was related to ranking for butterfly (22.4 ± 3.5 yrs, *r* = −0.29, *p* < 0.001), backstroke (21.6 ± 2.8 yrs, *r* = −0.27, *p* = 0.002), breaststroke (23.3 ± 3.1 yrs, *r* = −0.17, *p* = 0.045), and freestyle (22.2 ± 3.0 yrs, *r* = −0.20, *p* = 0.005) but not individual medley (24.0 ± 3.7 yrs, *r* = −0.25, *p* = 0.215).

### Regression Analysis

Using multiple linear regression, predictive values determined the 2018EC ranking based on ACV, number of races per year, and age. For pooled data of all strokes and distances, regression analysis explained 20% (*p* < 0.001) of total variance in the 2018EC ranking (Table 3). All predictors contributed significantly to the outcome variable (*p* < 0.001). Number of races had the largest effect (β = −0.34), followed by age (β = −0.19), and ACV (β = −0.16).

**Table 3:** Multiple linear regression analysis for annual variation in competition performance (ACV), number of races per year (Races), and age.

	**Regression model**				**Regression coefficients**			
	**Entries**	**R square**	***F*-value**	***P*-value**		**Beta**	***T*-value**	***P*-value**
All strokes and distances	752	0.20	*F*_(3, 748)_ = 62	*P* < 0.001	ACV	−0.16	*T* = −5	*P* < 0.001
					Races	−0.34	*T* = −10	*P* < 0.001
					Age	−0.19	*T* = −6	*P* < 0.001
Butterfly	152	0.28	*F*_(3, 148)_ = 19	*P* < 0.001	ACV	−0.19	*T* = −3	*P* = 0.008
					Races	−0.37	*T* = −5	*P* < 0.001
					Age	−0.30	*T* = −4	*P* < 0.001
Backstroke	132	0.30	*F*_(3, 128)_ = 19	*P* < 0.001	ACV	−0.15	*T* = −2	*P* = 0.049
					Races	−0.45	*T* = −6	*P* < 0.001
					Age	−0.31	*T* = −4	*P* < 0.001
Breaststroke	133	0.39	*F*_(3, 129)_ = 27	*P* < 0.001	ACV	−0.06	*T* = −1	*P* = 0.416
					Races	−0.59	*T* = −8	*P* < 0.001
					Age	−0.09	*T* = −1	*P* = 0.204
Freestyle	197	0.37	*F*_(3, 193)_ = 37	*P* < 0.001	ACV	−0.13	*T* = −2	*P* = 0.024
					Races	−0.53	*T* = −9	*P* < 0.001
					Age	−0.15	*T* = −3	*P* = 0.008
Individual medley	27	0.17	*F*_(3, 23)_ = 2	*P* = 0.228	ACV	0.08	*T* = 0	*P* = 0.687
					Races	−0.34	*T* = −2	*P* = 0.099
					Age	−0.18	*T* = −1	*P* = 0.389
50 m	60	0.47	*F*_(3, 56)_ = 16	*P* < 0.001	ACV	−0.25	*T* = −2	*P* = 0.027
					Races	−0.42	*T* = −4	*P* < 0.001
					Age	−0.29	*T* = −3	*P* = 0.005
100 m	77	0.45	*F*_(3, 73)_ = 20	*P* < 0.001	ACV	−0.17	*T* = −2	*P* = 0.056
					Races	−0.59	*T* = −7	*P* < 0.001
					Age	−0.20	*T* = −2	*P* = 0.024
200 m	60	0.31	*F*_(3, 56)_ = 9	*P* < 0.001	ACV	0.02	*T* = 0	*P* = 0.894
					Races	−0.56	*T* = −5	*P* < 0.001
					Age	−0.02	*T* = 0	*P* = 0.837
400 m	35	0.29	*F*_(3, 31)_ = 4	*P* = 0.013	ACV	0.01	*T* = 0	*P* = 0.987
					Races	−0.54	*T* = −3	*P* = 0.001
					Age	−0.17	*T* = −1	*P* = 0.281
800 m	26	0.28	*F*_(3, 22)_ = 3	*P* = 0.063	ACV	−0.17	*T* = −1	*P* = 0.405
					Races	−0.07	*T* = 0	*P* = 0.749
					Age	−0.50	*T* = −3	*P* = 0.012
1,500 m	20	0.19	*F*_(3, 16)_ = 1	*P* = 0.315	ACV	−0.07	*T* = 0	*P* = 0.803
					Races	−0.13	*T* = −1	*P* = 0.617
					Age	−0.42	*T* = −2	*P* = 0.084

The regression model explained a significant proportion (*p* < 0.001) of total variance in the 2018EC ranking for butterfly (28%), backstroke (30%), breaststroke (39%), and freestyle (37%). Individual medley remained unexplained. For all strokes, number of races per year had the largest effect on ranking (β = −0.37 to −0.59). In addition, ACV and age contributed significantly to variation of the 2018EC ranking for butterfly, backstroke, and freestyle (refer to Table 3).

In regard to race distance, the regression model explained a highly significant proportion (*p* < 0.001) of variance in the 2018EC ranking for 50 m (47%), 100 m (45%), and 200 m races (31%). A significant proportion (*p* = 0.013) was also explained for 400 m (29%) but not 800 and 1,500 m races. Number of races showed the largest effect on the dependent variable for the 50, 100, 200, and 400 m races (β = −0.42 to −0.59). A significant age effect was evident for 50 m (β = −0.29) and 100 m races (β = −0.20). The ACV showed a significant effect on 50 m races only (β = −0.25).

## Discussion

The main findings of this study were that number of races per year had the largest effect on ranking at the 2018EC, followed by age and ACV. The regression model explained a significant proportion of the 2018EC rankings for butterfly (28%), backstroke (30%), breaststroke (39%), and freestyle (37%) but not individual medley. Regarding race distance, rankings for 50 m (47%), 100 m (45%), 200 m (31%), and 400 m races (29%) were explained, but not for 800 and 1,500 m races. Although, correlations were small to medium, higher ranked swimmers at the 2018EC showed a significantly greater number of races per year and larger ACV for all ten and nine consecutive years, respectively, prior to the 2018EC.

The recent development in swimming, with the introduction of new multi-stage events, such as the International Swimming League (ISL, 2019), in addition to the established World Cup races and season's main events, i.e., Europeans, World Championships, and Olympic games, increased number of races in the competition schedule (FINA, 2020a). Especially successful swimmers may be invited to and qualify for additional events. Indeed, the results of the present study showed a larger number of races per year for higher ranked swimmers at the 2018EC. With such a high number of races, a complete 2–4 week taper period is not feasible for every competition, as loss of training time would accumulate over the course of the season (Hellard et al., 2019). However, when training and competition load are matched and incorporated into a well-periodized season (Hellard et al., 2019), racing can be a very specific and intense way of training (Carrasco et al., 2007; Nugent et al., 2017) and aid physiological, technical, and psychological skill acquisition (Gould and Rolo, 2004; Carrasco et al., 2007; Meggs et al., 2016; Nugent et al., 2017; Ribeiro et al., 2017). Therefore, races of minor importance should not be neglected but used for training purpose although these unprepared and untapered races may lead to race results off the swimmer's personal best and may increase performance variation. However, higher ACV was related to better 2018EC ranking and unsuccessful races did not seem to hamper peak performance at the season's main event.

In addition to physiological, psychological factors may affect number of races per year. Highly talented and successful athletes are associated with a high intrinsic motivation (Issurin, 2017), task orientation, and a growth mindset (Cervello and Santos-Rosa, 2001; Dweck, 2009; Dweck and Yeager, 2019). The success these swimmers experience in competition may further motivate them for race participation (Weinberg, 1979) and help to explain the relation between higher ranking and greater number of races per year. Thus, lower ranked and less successful swimmers may also benefit from more opportunities to practice their race specific skills and prove themselves to others in competition (Mccormick et al., 2015). With the right assessment, less important races can be accepted as a challenge and help to develop the right mindset, build competition routine, and self-confidence, rather than fear and anxiety (Gould and Rolo, 2004). In particular, during adolescents, a larger number of races could provide learning opportunities to develop important psychological skills, such as dealing with defeat, coping with expectations, and developing resilience (Sarkar et al., 2015; Meggs et al., 2016; Burns et al., 2019). As higher ranked swimmers at the 2018EC swam more races for all 10 years prior to the championships, we conclude that a high number of races, when differing between so-called learning competitions and major events, may support the development of young swimmers.

In the present study, higher ranked athletes at the 2018EC were older compared to their lower ranked peers. Ericsson and co-workers proposed the concept of deliberate practice, where a certain amount of training, i.e., ~10,000 h within 10 yrs, is required to achieve elite performance (Ericsson et al., 1993; Ericsson and Harwell, 2019). While there is a heated debate on total variance explained within the multifactorial talent model (Macnamara et al., 2014; Ericsson and Harwell, 2019), quality and amount of practice accumulated with age seems to impact long-term athlete development in swimming, in addition to genetics, economies, and available support (Tucker and Collins, 2012; Breitbach et al., 2014). More detailed analyses of the present data showed that age was correlated to ranking in butterfly, backstroke, breaststroke, and freestyle but not to individual medley ranking. This may be explained by young age at peak performance for individual medley (Allen et al., 2014; Dormehl et al., 2016), the early specialization in swimming (Moesch et al., 2011; Feeley et al., 2016), or performance density that may also be different between swimming strokes of the pool events (Baldassarre et al., 2017). However, differences in performance density between swimming strokes warrant further investigation, in particular its effect on swimmers' age.

Previous studies showed that performance of elite swimmers peaked at a mean age of 24.2 ± 2.1 yrs with a 2.6 ± 1.5 yrs window of peak performance (Allen et al., 2014). While age of peak performance decreased with increased race distance (Wolfrum et al., 2013; Allen et al., 2014), the present study also showed a smaller effect of age on ranking with increased distance in 50–200 m freestyle races. This finding is in contrast to other endurance sports, in which age of peak performance increases with longer distances (Allen and Hopkins, 2015). From a physiological perspective, the aerobic capacity and movement economy is built up over years (Zaryski and Smith, 2005), increasing far beyond the age of 22.9 ± 2.2 yrs that is established as age of peak performance in male long-distance pool swimmers (Allen et al., 2014). However, swimming, in particular long-distance pool events, is generally perceived to be mentally tough, with psychological factors dominating drop-out numbers (Monteiro et al., 2017). Therefore, motivational rather than physiological aspects may affect age of peak performance. Previous studies showed, that participation at (Junior-) World Championships are important success factor for upcoming competitions (Yustres et al., 2017, 2019) and that success is a great motivator to keep up the hard work needed for training and competition at a high level (Weinberg, 1979). However, early success at junior level is no guarantee for success at elite age (Barreiros et al., 2014) and long-term athlete development systems, which vary depending on the nation, may affect age at peak performance (Ford et al., 2011; Lloyd et al., 2016). As the present study showed that higher ranked swimmers at the 2018EC were older, from an individual swimmer's perspective, peaking at a later age may be beneficial for elite age success. Swimmers who do not progress to (semi-)finals at European championships are encouraged to gain experience and maintain motivation for high-level training and competition (Yustres et al., 2017, 2019). As such, swimmers below age at peak performance may increase their success chances for later championship participations.

### Methodological Considerations

Higher ranked swimmers at the 2018EC may participate in more races, due to racing in the heats, semi-finals, and finals (Pyne et al., 2004; Trewin et al., 2004). Additionally, at a senior elite level, more successful swimmers may be more frequently invited to meets and qualify for more competitions, i.e., International Swimming League, World Cup, etc. (ISL, 2019; FINA, 2020a). However, qualification trials for the European championships are fully competitive with no wild cards granted (LEN, 2020a). Therefore, lower ranked swimmers participate in heats, semi-finals, and finals at qualification, national, and other international competitions throughout the season and may experience a similar number of options for race participation. From a practical perspective, international swimmers may compete successfully in more than one swimming stroke, which would contribute to an increased number of races (IOC, 2016b; LEN, 2020b). However, skill transfer between swimming strokes and the effect on number of races per year, long-term athlete development, and competition success warrant further investigations.

The present study is limited to European swimmers. Despite statistical significance, only small to medium correlations for number of races and ACV were found. However, the present study represents the initial approach to identify novel success factors. Future studies should quantify the effect of number of races and ACV in the multi-dimensional model of talent development and progression, which involves psychological, economical, genetic, and social factors (Tucker and Collins, 2012; Breitbach et al., 2014; Gullich and Emrich, 2014; Macnamara et al., 2014; Lloyd et al., 2015a,b; Malina et al., 2015; Ericsson and Harwell, 2019). For the practical application, competition data are feasible to derive and provide objective data for talent development and identification. For the present investigation, data were derived with no information on pacing strategies applied in the races. Better swimmers commonly pace through the heats and semi-finals in order to save energy for the finals, which may have increased ACV for the higher ranked swimmers (Pyne et al., 2004; Trewin et al., 2004). Future studies should take race analyses and applied pacing strategies into account when investigating performance variation.

## Conclusion

While talent development undoubtedly involves multiple factors, i.e., genetics, economies, available support, quality and amount of practice accumulated with age (Tucker and Collins, 2012; Ericsson and Harwell, 2019), the present study investigated ACV, number of races per year, and age as potential success factors for international swimming competitions. Higher ranked swimmers were older than their lower ranked peers. Therefore, young swimmers below age of peak performance who do not progress to (semi-)finals at European championships are encouraged to continue competing at a high-level and benefit from success chances that increase with age. As higher ranked competitors swam more races per year, with a greater ACV across the 10 years investigated, future studies should quantify the effect of these factors on the multi-dimensional model of long-term athlete development and progression (Tucker and Collins, 2012; Breitbach et al., 2014; Gullich and Emrich, 2014; Macnamara et al., 2014; Lloyd et al., 2015a,b; Malina et al., 2015; Ericsson and Harwell, 2019). From a practical perspective, race results far off the personal best during the course of the season did not impair success at the season's main event, where athletes were fully tapered and prepared. The larger number of races per year that are swum throughout the career of successful swimmers could provide mental learning opportunities and race specific technical and physiological adaptations. In particular, young swimmers could use races of less importance for these learning opportunities which may increase success later on in their career. Coaches should assess unsatisfactory race results with care and help athletes to build competition routine and self-confidence, rather than fear and anxiety (Gould and Rolo, 2004). While participation in competition requires time and energy for traveling (Calleja-Gonzalez et al., 2020), number of races (entries) at a given number of competition could be increased in order not to compromise time for practice. Future research needs to compare number of competitions and number of races (entries) per year regarding success at the season's main event.

## Data Availability Statement

All data are available on the publicly accessible database https://www.swimrankings.net/ and can be retrieved by any user.

## Author Contributions

D-PB and MR: conception of the experimental design. D-PB, IL, EM, PS, DB, and MR: data collection. D-PB, IL, SH, EM, PS, DB, and MR: data analysis and reading and approving final version of the manuscript. D-PB, SH, and MR: data interpretation. D-PB: preparing the manuscript. IL, SH, EM, PS, DB, and MR: critically revising the manuscript. All authors contributed to the article and approved the submitted version.

## Conflict of Interest

The authors declare that the research was conducted in the absence of any commercial or financial relationships that could be construed as a potential conflict of interest.
